# Supplementary material for: Lymphoid Organ Proteomes Identify Therapeutic Efficacy Biomarkers Following the Intracavitary Administration of Curcumin in a Highly Invasive Rat Model of Peritoneal Mesothelioma
Source: Int J Mol Sci. 2021 Aug 9;22(16):8566. doi: 10.3390/ijms22168566 (PMC8395293; doi:10.3390/ijms22168566)
Supplement: Supplementary file 1 [file ijms-22-08566-s001.zip › ijms-1275619-supplementary.pdf]

Supplementary Materials

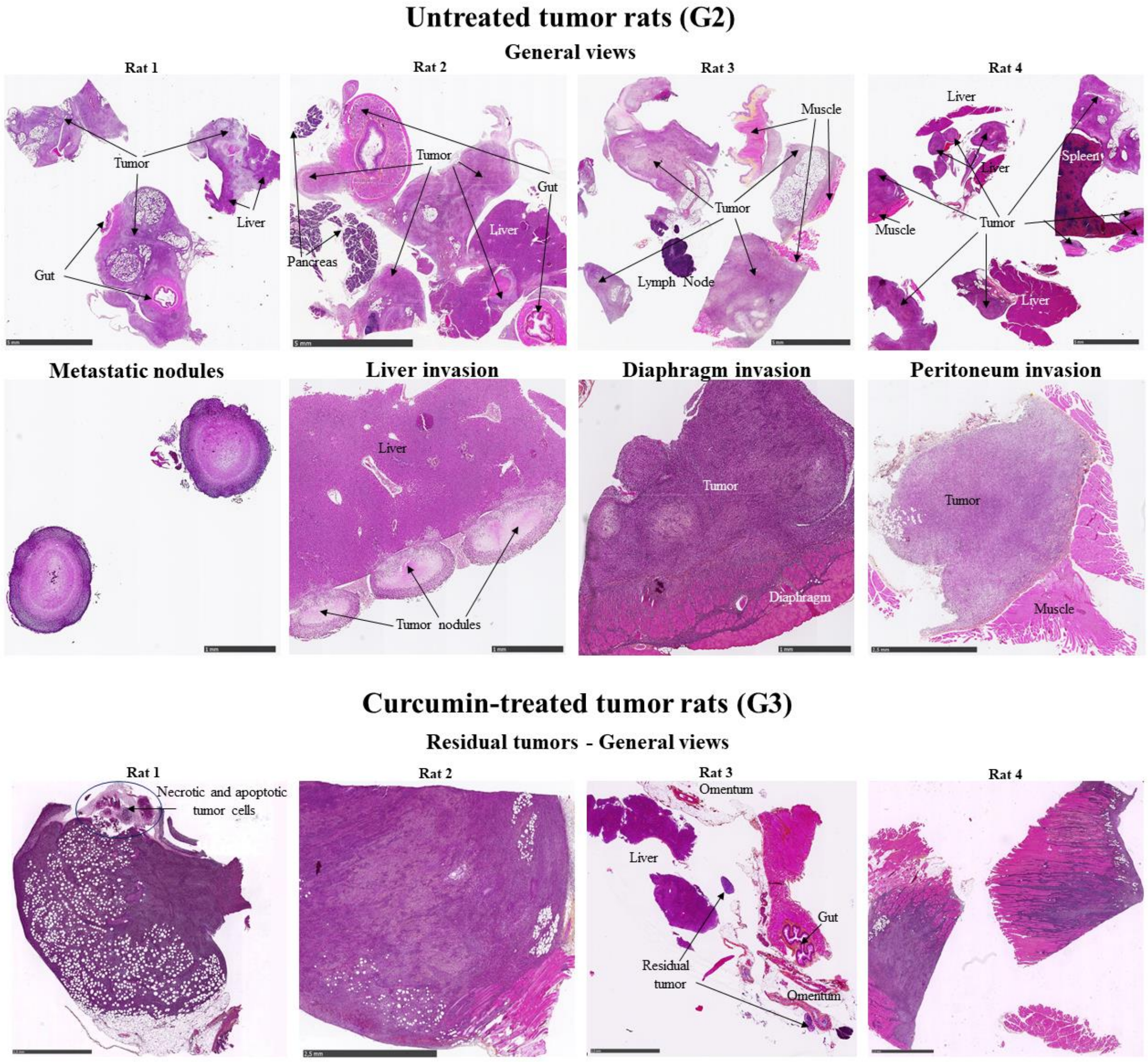

Figure S1. General views and histological features of M5-T1 tumors collected in each rat of the G2 and G3 groups. HPS staining, the scale bars are indicated at the bottom of each photograph.

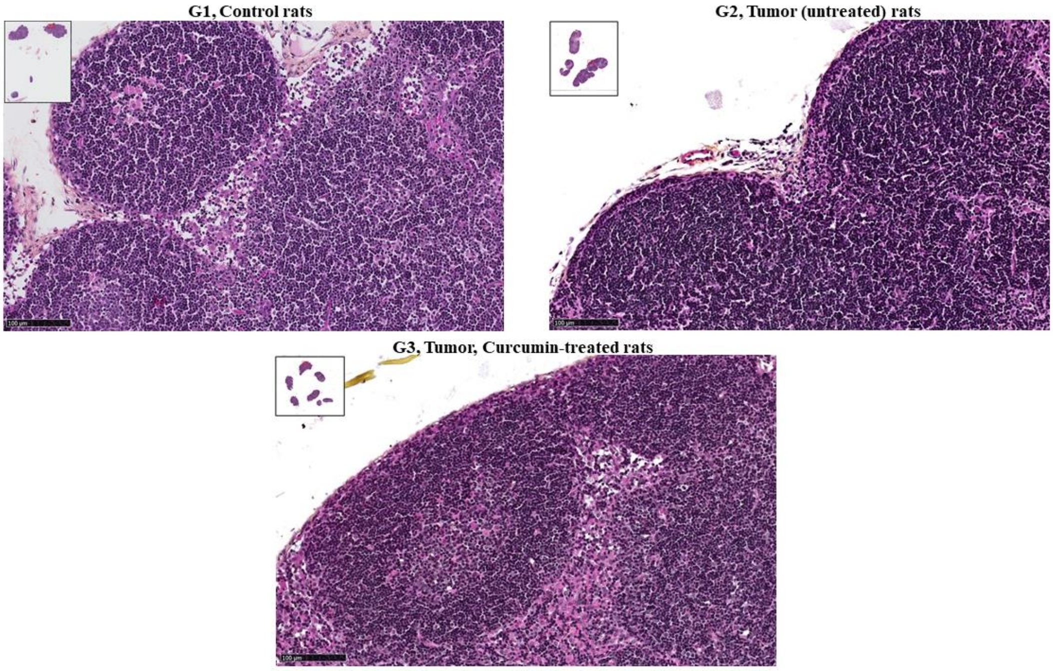

Figure S2. Representative examples of the histological features of mesenteric lymph nodes from the three groups of rats. HPS staining, ×200, the scale bars represent 100 µm. Inserts show general views of the whole samples with red rectangles representing locations of selected areas.

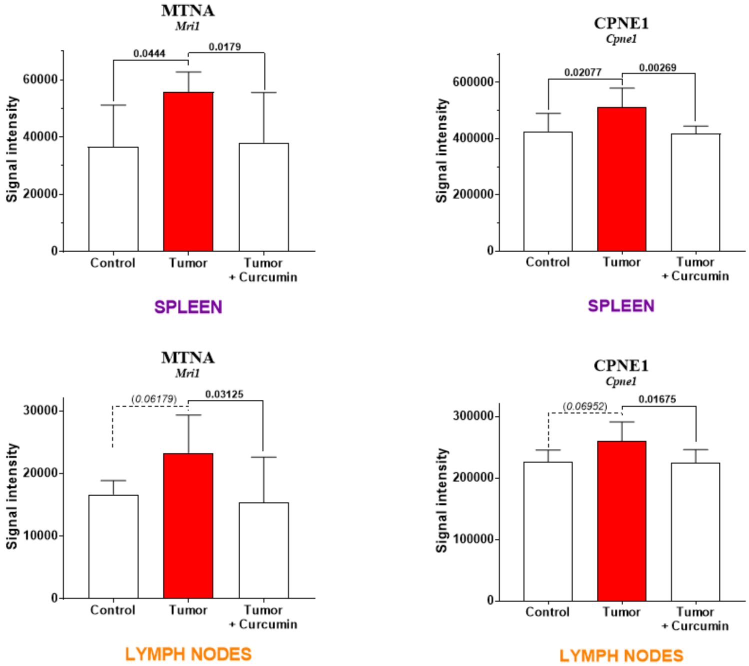

Figure S3. Additional proteins of interest (supplement to Figure 5) exhibiting common profiles of abundance changes in spleen and mesenteric lymph nodes. Dashed lines represent tendencies, with corresponding *p* values in italics.
